# PReS-FINAL-2197: Teenage boy suffering from diabetes mellitus type 1 and heterozygous Familial Mediterranean Fever: a case report

**DOI:** 10.1186/1546-0096-11-S2-P187

**Published:** 2013-12-05

**Authors:** AN Olivieri, MF Gicchino, C Granato, A Mauro, A Mellos

**Affiliations:** 1 Pediatrics, Second University of Naples, Naples, Italy

## Introduction

It is known that type 1 diabetes mellitus (type 1 DM) maybe associated with other autoimmune diseases, such as autoimmune thyroid disease (ATD), celiac disease (CD) and Addison's disease (AD). We report the case of a patient with type 1 DM associated with familial mediterranean fever (FMF). It is the third association of these two diseases described in the medical literature to our knowledge so far.

## Objectives

Our aim is to emphasize that FMF must be taken into consideration as a possible disease associated with type 1 DM in the presence of suggestive findings.

## Methods

A 13 year old boy already suffering from diabetes mellitus type 1 since the age of 4 years and 3 months, came to our attention because of the presence of periodic fever associated with abdominal pain, oral ulcers, chest pain and diffuse arthralgia. The fever appeared every 15-30 days with peaks that reached 40°C and lasted 24-48 hours. Blood tests (complete blood count, blood culture, serum immunoglobulins, viral serology, biochemical profile), instrumental examinations (ultrasound of the abdomen and chest x-ray) and the rest of laboratory investigations (culture of throat swab, stool examination and urinalysis) were normal in the interval between febrile episodes, but during the attacks revealed an increase in inflammatory markers (ESR 60 mm/1 h, CRP 3.7 mg/dl). For the clinical suspicion of FMF we requested that the genetic investigation was performed.

## Results

The molecular analysis of the nucleotide sequence of exon 2 of MEFV gene, performed in genomic DNA extracted from peripheral blood leukocytes, showed the mutation c.442G> C (p.E148Q) in the heterozygous state. The colchicine therapy, started 20 months ago at a dose of 1 mg/day, is well tolerated and has determined the immediate disappearance of the symptoms so far. Renal biopsy, performed at 14 years and 6 months of age because of persistent proteinuria, showed the absence of amyloidosis but a slight and irregular thickening of the lamina densa of some glomerular capillaries presumably due to diabetes. The serological tests have ruled out so far CD, AD, ATD and connective tissue diseases (CTD). Until now, the eye examination did not detect the presence of iridocyclitis.

## Conclusion

The coexistence of FMF and type 1 DM is a very rare finding. FMF heterozygotes tend to have a milder course of the disease and are less prone to experience new clinical manifestations than homozygotes. Moreover, at puberty, their symptomatology could disappear allowing to cease colchicine without relapses or an increase of inflammatory markers. Recently, a case of simultaneous protracted febrile myalgia syndrome (PFMS) preceded by diabetic ketoacidosis (DKA) has been described for which the authors have suggested that DKA-associated cytokine release could be a predisposing factor or a trigger for FMF-associated PFMS. Conversely, it was hypothesized that the immune dysregulation in FMF could be involved in the autoimmune mechanism that leads to type 1 DM. Finally, a prolonged follow up is needed to verify the long-term necessity of colchicine for our patient and further studies are required to reveal any possible shared molecular mechanisms that are responsible for these two diseases.

## Disclosure of interest

None declared.

